# Hybrid Horizons: Screening Hybridisation Through Nuclear Environmental DNA

**DOI:** 10.1111/1755-0998.70134

**Published:** 2026-05-04

**Authors:** Daniel Zumel, Emilie Didaskalou, Tijana Vučić, Milena Cvijanović, Ana Ivanović, Maja Ajduković, Ben Wielstra, Anagnostis Theodoropoulos, Kathryn Stewart

**Affiliations:** ^1^ Institute of Environmental Sciences Leiden University Leiden the Netherlands; ^2^ Institute of Biology Leiden University Leiden the Netherlands; ^3^ Naturalis Biodiversity Center Leiden the Netherlands; ^4^ Faculty of Biology, Institute of Zoology University of Belgrade Belgrade Serbia; ^5^ Institute for Biological Research “Siniša Stanković”, National Institute of the Republic of Serbia, Department of Evolutionary Biology University of Belgrade Belgrade Serbia

**Keywords:** crested newts, KASP genotyping, non‐invasive monitoring, nuclear eDNA, proof‐of‐concept, *Triturus*

## Abstract

Effective monitoring of hybrid zones is essential for understanding evolutionary dynamics and mitigating species loss caused by human‐mediated hybridisation. Conventional methods rely on sampling numerous individuals, which is costly, time‐consuming and often impractical for rare or elusive species. Environmental DNA (eDNA) offers a promising alternative for locating hybrid zones but requires the detection of nuclear eDNA, which is typically scarce in natural ecosystems. While a few recent studies have successfully recovered sufficient nuclear eDNA to assess intraspecific variation, its application in hybridisation studies remains untested. This study provides the first empirical validation that nuclear eDNA can screen for hybrid populations. We present an eDNA‐based toolkit that employs Kompetitive Allele‐Specific PCR (KASP) to genotype a panel of species‐diagnostic unlinked nuclear SNPs without sequencing, mapping end‐point fluorescence to a hybrid index that reflects ancestry levels at the population scale. In mesocosms housing different combinations of individuals from two crested newt species ( *Triturus ivanbureschi* and *T. macedonicus* ) and their captive‐bred F1 hybrids, we compared eDNA‐derived ancestry estimates with genotypes obtained from skin swabs of the same individuals placed in the mesocosms. eDNA‐based ancestry estimates showed strong concordance with individual genotypes across two eDNA sampling concentrations. This approach represents a promising non‐invasive, fast and cost‐efficient screening tool, qualities that make it well suited to locate and track putative hybrid zones and a scalable complement to conventional sampling for biodiversity monitoring and conservation.

## 1 Introduction

Hybridisation is a pervasive evolutionary process, the outcome of which depends on the strength and permeability of reproductive isolation (Hewitt 1988; Harrison and Larson 2014). Its evolutionary effects are manifested at the individual and population level, leading to the formation of hybrid populations with individuals of mixed ancestry (Barton and Hewitt 1985). By mediating interspecific gene flow, also called introgression, hybridisation can accelerate the input of genetic variation far beyond mutation, shaping both speciation and adaptation (Barton and Hewitt 1985; Harrison 1990; Stewart et al. 2016). Conversely, introgression can homogenise biodiversity when hybrids outperform parental species and even cause species loss through genetic swamping, if parental species differ in fitness (Woodruff and Gould 1987; Liou and Price 1994; Allendorf et al. 2001). Human activities are amplifying these dynamics by modifying habitats and spreading non‐native taxa (McFarlane and Pemberton 2019; Ottenburghs 2021), which erodes reproductive barriers (Crispo et al. 2011; Grabenstein and Taylor 2018) and threatens the genetic integrity of specific lineages (Butler 1994; Goka 1998; Largiadèr 2007; Curto et al. 2022; DeVos et al. 2023; Pujolar et al. 2023; Theodoropoulos et al. 2025). Despite the seemingly major role hybridisation plays in shaping historical and contemporary biodiversity globally, detecting instances of hybridisation in nature remains laborious, time‐consuming and expensive, diminishing our ability to truly understand its frequency, pervasiveness and potential biodiversity outcomes (Stewart and Taylor 2020).

Monitoring hybridisation remains difficult because hybridisation events can arise and spread quickly and are often cryptic (Meilink et al. 2015). It occurs most frequently between recently diverged species with often subtle morphological differences, making hybrids hard to detect and differentiate from parental species (Seehausen 2004; Mallet et al. 2007; Hinneberg et al. 2022). Conventional practice therefore requires collecting genetic material within known or suspected hybrid zones (areas where the ranges of parental species meet and hybridisation occurs) from many individuals sampled from many populations. This initial approach to sampling hybridisation simultaneously targets two primary objectives: (i) to map the geographic location and extent of the hybrid zone(s) by estimating ancestry at the population scale (calculated as the aggregate allele frequencies of all individuals collected at a locality) and (ii) to detect individual hybrids within populations and assess their concomitant fitness with respect to their parental counterparts. However, the individual‐based sampling involved in both aforementioned objectives can be intrusive, imperil vulnerable populations (Zemanova 2019; Schilling et al. 2022; Mathwin et al. 2024) and incur high costs for rare or elusive species (Thompson 2013; Draheim et al. 2015), limitations that are exacerbated during exploratory analyses of hybridisation in nature. Such constraints motivate new sampling strategies and innovative tools that could separate out these complementary objectives, to focus only on screening and mapping putative hybrid zones by estimating the ancestry of the populations, without the need to collect individuals or to detect individual hybrids. By doing so, these strategies could be applied on a large scale to more efficiently assess the frequency, geographic locality and extent of putative hybrid zones (Stewart and Taylor 2020), further accelerating research on hybridisation.

Environmental DNA (eDNA) sampling offers a promising alternative to conventional methods for advancing hybridisation research (Stewart and Taylor 2020). eDNA refers to the DNA molecules that organisms shed into the environment (Taberlet et al. 2012; Bohmann et al. 2014; Didaskalou et al. 2022), which can be collected to assess species presence and relative abundance (Foote et al. 2012; Dysthe et al. 2018; Ballini et al. 2024), but see Yates et al. (2019). This non‐invasive approach may also reduce costs compared to conventional sampling techniques (Mahon et al. 2013; Sigsgaard et al. 2016; Evans et al. 2017; Qu and Stewart 2019) and has been successfully applied to detect both elusive, rare, cryptic and non‐native species (Nester et al. 2020; Jeunen et al. 2022; Duarte et al. 2023; Ballini et al. 2024; James et al. 2024). However, most eDNA studies to date target mitochondrial DNA (mtDNA) (Piggott 2016; McCauley et al. 2024). MtDNA is haploid, nonrecombining, maternally inherited and introgression‐prone, properties that can mask the occurrence of hybridisation and the location of hybrid zones (Ballard and Whitlock 2004; Roos et al. 2011; Toews and Brelsford 2012; Burton et al. 2013; Pereira et al. 2016; Teske et al. 2018). Nuclear eDNA could offer a path forward.

Recent work shows that nuclear eDNA can be amplified and capture genetic variation at the population scale, a foundational necessity for detection of hybrid populations (Sigsgaard et al. 2020; Stewart and Taylor 2020). For instance, Andres et al. (2021) found a high correlation between microsatellite‐derived allele frequencies from both eDNA and tissue samples in round goby fish ( *Neogobius melanostomus* ). Similarly, Liu et al. (2024) used single nucleotide polymorphisms (SNPs) to detect fine‐scale spatial genetic structure in the cichlid *Astatotilapia calliptera* across the water column of a lake. Yet for hybridisation specifically, applications remain conceptual (Stewart and Taylor 2020; van Kuijk et al. 2025) and lack empirical validation. A practical method is Kompetitive Allele‐Specific PCR (KASP), a genotyping technique for biallelic SNPs that can generate sequencing‐free calls from end‐point clustering, based on fluorescence emission in two different fluorescence channels (FAM and HEX). Through the use of species‐diagnostic nuclear SNPs, KASP genotyping allows the identification of homozygous and heterozygous genotypes from individual samples (Semagn et al. 2014). KASP is rapid, with the capacity to genotype thousands of samples within a single day, cost‐effective when small SNP panels are used (approximately €0.3 per reaction), and scalable, permitting repeated genotyping or subsequent expansion of SNP panels (Wielstra et al. 2016). Prior eDNA implementations aimed at assessing species presence in eDNA samples containing a single target species indicated that the use of KASP for the detection of nuclear eDNA is technically feasible (Schabacker et al. 2020; van Kuijk et al. 2025). However, KASP has not yet been tested using eDNA samples in which both target species occur in syntopy (co‐occurrence without hybridisation) or from hybrid populations (scenarios characterised by the presence of both target alleles and, consequently, potential fluorescence emission in both channels). When this approach is applied to species pairs for which prior evidence indicates permeability of reproductive barriers, the simultaneous detection of fluorescence in both channels would be indicative of the presence of a hybrid population.

This study aims to evaluate whether nuclear eDNA provides sufficient signal to detect evidence of hybridisation at the population scale. We develop and validate an eDNA‐based toolkit to identify hybrid populations by estimating population‐level ancestry, a necessary first step in hybrid zone delineation. The Balkan crested newt *Triturus ivanbureschi* and the Macedonian crested newt *T. macedonicus* , which hybridise naturally in the Balkan Peninsula (Arntzen et al. 2014; Wielstra et al. 2017), serve as a model system. Using species‐diagnostic nuclear SNPs, genotyped through Kompetitive Allele‐Specific PCR (KASP), we assess whether fluorescence‐derived ancestry values from eDNA can accurately reflect the allele composition of populations containing pure individuals and F1 hybrids. The experiment is conducted in controlled mesocosms under different eDNA concentrations, and the recovery of eDNA signals is validated with skin swabs.

## 2 Materials and Methods

### 2.1 Mescosm Experiment

We collected individuals of *T. ivanbureschi* and *T. macedonicus* from natural populations and housed these at the Institute for Biological Research ‘Siniša Stanković’, National Institute of the Republic of Serbia (University of Belgrade, Serbia). The *T. ivanbureschi* were collected in 2014 from Zli Dol, Serbia (42.4166° N, 22.4500° E) and in 2022 from Brebevnica, Serbia (43.0332° N, 22.8663° E). The *T. macedonicus* were collected in 2015 at Ceklin, Montenegro (42.3500° N, 18.9833° E). We produced F1 hybrids through two successive experimental crosses conducted in 2016 and 2017. Rearing and breeding conditions are detailed in Vučić et al. (2022). All experimental animals were treated in compliance with the European directive (see Ethics Statement section for more information). In total, we selected 9 adult *T. ivanbureschi* , 9 adult *T. macedonicus* and 18 adult F1 hybrids for our experiment. We took skin swabs from all 36 individuals to confirm their genotypes and recorded body mass to assess its effect on eDNA contribution.

For eDNA analysis, we collected water samples from plastic containers filled with 6 or 30 L of dechlorinated tap water, either housing animals or serving as negative field controls. To ensure sterilisation without negatively affecting the health of the newts, we decontaminated all containers with a 0.1% household bleach solution. We then thoroughly rinsed containers with tap water to remove any residual bleach and subsequently filled them to the top with tap water. We emptied the containers and refilled them with fresh tap water left to dechlorinate for approximately 24 h before introducing the newts.

**FIGURE 1:**
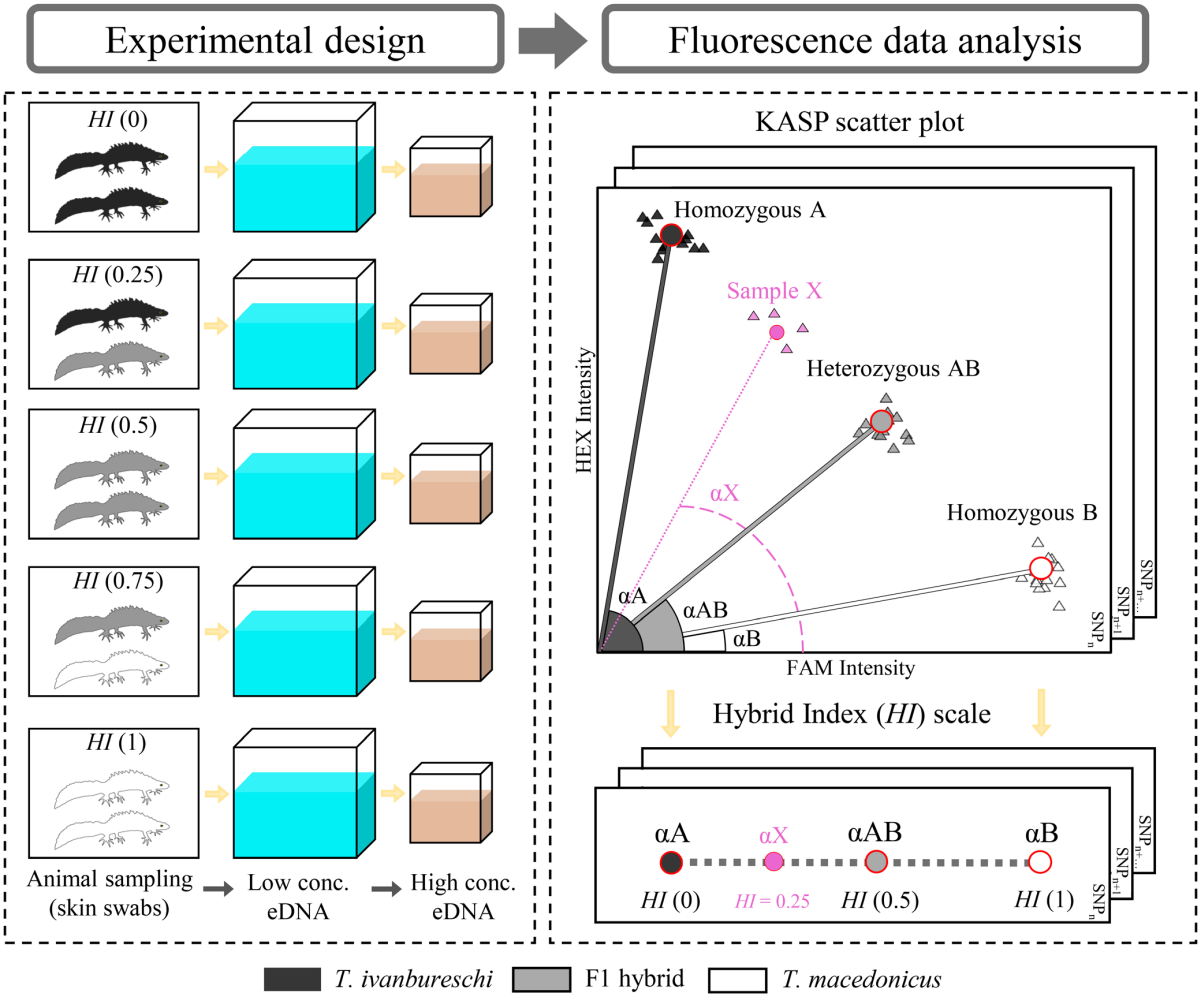
Experimental design and framework for converting fluorescence signals to hybrid index (*HI*). Left panel: Five genotypic profiles of *Triturus* newts, based on the expected value of *HI* according to their allelic proportions: *HI* (0) (two *T. ivanbureschi* ), *HI* (0.25) (one *T. ivanbureschi* + one F1 hybrid), *HI* (0.5) (two F1 hybrids), *HI* (0.75) (one *T. macedonicus* + one F1 hybrid), and *HI* (1) (two *T. macedonicus* ). Each population undergoes two sequential eDNA sampling regimes (low concentration [conc.] eDNA in blue; high concentration eDNA in brown). Right panel: Genotype centroids (A, AB, B) per SNP (*n* = 9) accommodate SNP‐specific primer competition, with the angle from the origin for each centroid (αA, αAB, αB; 0° along FAM, 90° along HEX) mapped linearly onto the *HI* scale (*HI* = 0 at αA, 0.5 at αAB, 1 at αB). Each black square denotes one SNP. A pink exemplar illustrates the computation: The centroid across four replicates is identified, its angle αX is measured, and αX is mapped onto the *HI* scale.

Each container held a single experimental unit, which we refer to as a population. Each population consisted of a pair of individuals. Populations were assigned to genotypic profiles based on their Hybrid Index (*HI*), defined as the proportion of *T. macedonicus* alleles in the population (*HI* range 0–1; 0 = only *T. ivanbureschi* alleles, 1 = only *T. macedonicus* alleles). The five genotypic profiles were: *HI* (0): two *T. ivanbureschi* ; *HI* (0.25): one *T. ivanbureschi* + one F1 hybrid; *HI* (0.5): two F1 hybrids; *HI* (0.75): one *T. macedonicus* + one F1 hybrid; *HI* (1): two *T. macedonicus* (see Figure 1). Each genotypic profile was represented by multiple biological replicates, meaning independent populations (separate containers) with the same genotypic composition. Specifically, each profile had three replicate populations, except *HI* (0.5), which had six replicate populations, resulting in 18 populations in total. To minimise biological variance in shedding rates in populations with mixed genotypes (*HI* = 0.25 and *HI* = 0.75), only male individuals were used for these specific experimental units.

To test the effect of eDNA concentration on the quantification of *HI*, we subjected each population to two subsequent sampling regimes (Figure 1). First, individuals remained in a container with 30 L of water for 24 h, after which we removed them and filtered 400 mL of water, representing 1.33% of the total water volume. Next, we transferred the animals to a smaller container with 6 L of water for 24 h and then filtered 400 mL or 6.66% of the total volume of water. Following a cursory mixing with a sterile collection cup and assuming a homogeneous distribution of eDNA molecules in the water in general (Thomsen et al. 2012; Evans et al. 2017; Sakata et al. 2021), sampling in 6 L container (hereafter the ‘high concentration’ sampling regime) should yield approximately five times as many eDNA molecules compared to sampling in 30 L container (hereafter the ‘low concentration’ sampling regime). We maintained two additional containers of 30 and 6 L without animals for the duration of the experiment as negative field controls. In all cases, we sampled water from each container via sterile 500 mL containers and filtered water through 0.45 μm polyethersulphone (PES) membrane filters placed in sterilised Nalgene reusable filter units (Thermo Fisher) connected to a pump. We removed and folded filters using sterile tweezers and preserved them in 700 μL Longmire's solution in 2 mL Eppendorf tubes, thereafter shipped them on dry ice to Leiden University (The Netherlands), where we stored them at −20°C until DNA extraction. We sterilised all eDNA sampling equipment with 10% bleach, rinsed it in sterile, deionised water and dried it prior to use and between sampling.

### 2.2 DNA Extraction and Genotyping

We extracted DNA from skin swabs following a salt extraction protocol described by Sambrook and Russell (2001), using the Wizard Genomic DNA Purification Kit (Promega, Wisconsin, USA). For eDNA samples, we extracted DNA using the QIAGEN DNeasy Blood and Tissue Kit, following the recommendations of Spens et al. (2017), with the addition of the use of QIAshredder columns as described in Goldberg et al. (2011).

We genotyped a panel of nine unlinked nuclear SNP markers located in the 3′ UTR regions of protein‐coding genes, previously designed as species diagnostic for *T. ivanbureschi* and *T. macedonicus* and tested for linkage (Arntzen et al. 2018; Table S1). We performed genotyping using the KASP genotyping system (LGC genomics, UK) at the SNP genotyping facility of the Institute of Biology, Leiden University. KASP is a fluorescence‐based PCR variant that uses two SNP‐specific forward primers and a common reverse primer. Each forward primer contains a unique tail sequence for a Förster Resonance Energy Transfer (FRET) cassette labelled with either FAM or HEX dye. Thus, detecting FAM, HEX or both fluorescence signals allows for the determination of homozygous or heterozygous genotypes (Semagn et al. 2014). Here, we extend the applications of KASP to determine not only the presence of alleles, but also their population proportions using fluorescence readings (see 2.3 Data Analysis below for details).

For the KASP reactions, we dispensed 3 μL of 1:5 diluted template into 384‐well plates; this volume was chosen to maximise pipetting precision while reducing PCR inhibitors. Following LGC recommendations, we dried templates at 55 C for 1 h to ensure the final reaction volume achieved the correct reagent concentrations. Once dried, we added KASP‐TF V4.0 2X Master Mix, assay (comprising KASP primers) and water in proportions of 50%, 1.4% and 48.6%, respectively, to a final volume of 3 μL. We ran four PCR reactions per sample and marker combination. The PCR consisted of an initial step of 15 min at 94°C, followed by 51 cycles of 30 s at 94°C and 60 s at 61°C. We measured fluorescence on a PHERAstar plate reader after 39, 42, 45, 48 and 51 cycles. According to LGC recommendations, a maximum of 48 amplification cycles is advised; however, to ensure sufficient amplification of low‐template samples, we extended the amplification to 51 cycles. Since excessive cycles can lead to primer–dimer formation and distort fluorescence measurements (Nolan et al. 2020), we included at least four no‐template controls (NTCs) per plate, in addition to four negative controls with water instead of template. We also added one positive control per species and SNP to verify assay specificity and ensure consistent allele calling. From the five measurements (after 39, 42, 45, 48 and 51 cycles), we selected the one corresponding to the maximum number of amplification cycles that still showed no detectable dimers in NTCs and negative controls.

### 2.3 Fluorescence Analysis

We examined fluorescence readings to assess the detection of homozygous and heterozygous genotypic profiles in the populations and to minimise technical or biological bias. First, we excluded any PCR signal falling within a low‐fluorescence triangular region in the (FAM, HEX) space, defined by the vertices (0,0), (2,0) and (0,2), as this area was dominated by background noise and unsuccessful amplifications. Second, for the remaining reactions, we averaged fluorescence values per sample and SNP (up to four replicates) to reduce stochastic variation, thereby obtaining a more precise and representative signal for each sample–SNP combination. Third, we verified the genotypes of the animals placed in containers, whose DNA was obtained from skin swabs. For all SNPs, we calculated the centroid for each genotype ( *T. ivanbureschi* , *T. macedonicus* , F1 hybrid) using the median fluorescence of all samples with that expected genotype based on the geographic origin of the animals. We then measured Euclidean distance between each sample and its expected genotype centroid. In cases where an individual's signal was closer to a centroid other than the one expected from its collection locality metadata, we flagged it as a ‘mismatched genotype’, indicating potential genotyping error or mislabelling. To avoid propagating uncertainty and to ensure proper validation of the eDNA data, we excluded these genotypes together with their corresponding environmental samples.

To assess how well our eDNA samples could differentiate populations with mixed ancestry, we mapped KASP end‐point fluorescence to a hybrid index ‘*HI*’ (range 0–1). We first defined genotype centroids A ( *T. ivanbureschi* ), AB (F1 Hybrid; allelic ratio 1:1) and B ( *T. macedonicus* ) and calculated the Euclidean distance between each animal sample and its assigned centroid. We then classified samples with distances ≥ median + 3 × MAD (median absolute deviation) as outliers and excluded them to avoid inflating variance in centroid estimation (sensu Leys et al. (2013)). Next, we calculated each centroid's angle, αA, αAB and αB, from the origin (FAM, HEX = 0,0), with 0° along the FAM axis and 90° along the HEX axis. In KASP assays, each diagnostic allele is detected in a separate fluorescence channel (HEX for allele A, FAM for allele B); therefore, homozygotes should cluster at αA = 90° and αB = 0°. In practice, however, homozygotes commonly show low residual fluorescence in the opposite channel, likely due to imperfect quenching or non‐specific amplification, which shifts αA slightly below 90° and αB slightly above 0°. Moreover, unequal primer competition can induce SNP‐dependent displacement of the heterozygous cluster (LGC Genomics 2015), leading to variation in αAB across SNPs, meaning the heterozygous cluster does not always align at the theoretical 45° angle (representing a 1:1 ratio of fluorescence), but instead shifts depending on the specific primer efficiency of each marker. To capture the FAM/HEX ratio while minimising SNP‐dependent technical variation, we calibrated fluorescence readings across SNPs using piecewise linear interpolation along the arc defined by the three centroids, setting *HI* = 0 at αA, *HI* = 0.5 at αAB and *HI* = 1 at αB. We used the same procedure to obtain *HI* values for all individual animal and eDNA samples, mapping the angle of each sample onto the *HI* scale. To preserve linearity, we did not truncate samples with HEX values above A or FAM values above B, allowing minor deviations beyond the [0,1] range (Figure 1).

### 2.4 Statistical Analysis

To evaluate the consistency between the expected ancestry derived from individual genotypes and the observed ancestry estimates obtained via eDNA, we compared observed (*HI*_obs_) and expected (*HI*_exp_) *HI* values, with *HI*_exp_ computed from the verified allele proportion of each population. As Shapiro–Wilk tests rejected the normality of *HI*_obs_ deviations, we used non‐parametric methods throughout.

We assessed the reliability of individual SNPs by calculating the systematic bias of each marker using samples from animal swabs with confirmed genotypes. We calculated this bias as the median deviation (*HI*_obs_–*HI*_exp_) and obtained a 95% bootstrap confidence interval for this median. We retained SNPs only when the entire confidence interval fell within ±0.05 *HI* units. We set this threshold, corresponding to a 5% allowable deviation on the *HI* scale, as a conservative tolerance level to exclude SNPs with deviations that could impact *HI* estimates while accounting for experimental variability.

To assess the correlation between expected and observed values, for each sampling regime (low concentration and high concentration eDNA), we evaluated the association between *HI*_obs_ and *HI*_exp_ via Spearman's rank correlation. Next, to test whether sampling regime altered how well the observed values matched expectations, we fitted a permutation‐based ANCOVA (*HI*_obs_ ∼ *HI*_exp_ × sampling regime).

To evaluate whether the precision of *HI* estimation was associated with the population genotypic profile, we applied Kruskal–Wallis tests across the five evaluated profiles (*HI* = 0, 0.25, 0.5, 0.75, 1) within each sampling regime. We then identified genotypic profiles with systematic deviations from *HI*_exp_, using one‐sample Wilcoxon signed‐rank tests, with Holm correction. Next, we assessed whether variance was homogeneous among genotypic profiles, using pairwise Fligner–Killeen tests with Holm adjustment.

To test the performance of the toolkit at the population scale, we averaged *HI*_obs_ across SNPs per sample to obtain a single overall *HI* value. This allowed us to evaluate the precision of the *HI* estimation via two Bland–Altman analyses (Bland and Altman 1986). First, to assess sampling regime‐specific accuracy and precision, we calculated *HI*_obs_–*HI*_exp_ for each population within each sampling regime. We summarised these deviations by the mean bias and the 95% limits of agreement (LoA = mean±1.96 SD), which represent the interval around the mean bias within which ~95% of the deviations are expected to fall. Second, to assess reproducibility between sampling regimes, we computed the difference in *HI* estimates for each population sampled under both sampling regimes (*HI*_low conc–_ *HI*_high conc_.), also reporting the mean bias and 95% LoA.

Finally, to evaluate whether unequal individual contributions to the eDNA pool could bias estimates in populations containing individuals of different genotypes, we recomputed *HI*_exp_ for those populations using biomass correction of individual expected *HI* values. We then compared, for *HI* (0.25) and *HI* (0.75), the absolute deviations |*HI*_obs_–*HI*_exp_| under uncorrected versus biomass‐corrected values, using paired Wilcoxon signed‐rank tests with Holm correction.

We performed all calculations in R v4.4.1 (R Core Team 2024) using *dplyr* (Wickham 2015) for data manipulation, *stats* (R Core Team 2024) for correlation, rank‐based and variance tests, *lmPerm* (Wheeler and Torchiano 2025) for permutation‐based ANCOVA and *ggplot2* (Wickham 2016) for graphical outputs.

## 3 Results

We performed a total of 3114 PCR reactions: 1296 from skin swabs used for validation, 1296 from eDNA samples, 72 field negative controls, 432 lab negative controls and 18 positive controls (Table S2). Of these, we excluded 116 reactions from subsequent analysis because their fluorescence fell within the predefined low‐fluorescence area, including 63 reactions from animal swabs (4.86% of the validation dataset) and 53 from eDNA samples (4.08% of the eDNA dataset). These PCRs with low signal were removed prior to calculating average fluorescence values for each sample–SNP combination (up to four replicates).

Genotype verification of the 36 animals used in the experiment revealed that individual M12A yielded unexpected heterozygous signals across all markers, indicating that this animal had been mislabelled as *T. ivanbureschi* and was in fact an F1 hybrid. Therefore, we excluded it from further analysis. We also removed the eDNA samples from the two containers in which this animal was placed (one from each sampling regime), both assigned to *HI* (0.25), thereby reducing this genotypic profile to two biological replicates instead of three and the total number of containers from 36 to 34. Apart from this individual, we detected and excluded a total of 15 mismatched genotypes out of 315 calls (35 animals across 9 SNPs) in the animal swabs (4.76%), distributed among eight individuals. Of these mismatches, 11 corresponded to F1 hybrids for which the observed genotype was homozygous, and the remaining four corresponded to homozygous individuals for which the observed genotype was heterozygous. As these genotypes could not be reliably verified, and consequently, the resulting eDNA samples cannot be validated, these mismatches led to the exclusion of 30 calls from a total of 306 (34 containers across 9 SNPs) from the eDNA dataset (Table S3).

In addition, we detected fluorescent signals outside the predefined low‐fluorescence area in the negative field control of the high concentration eDNA sampling regime (empty 6 L container). These signals were observed in 23 of 36 PCRs and for all SNPs, suggesting possible contamination. The negative field control of the low concentration eDNA sampling regime (30 L container) did not yield any signal for any marker, nor did any of the other negative laboratory controls.

The calculation of genotype centroids to obtain per‐SNP angles for subsequent SNP calibration required the exclusion of 34 genotypic outliers out of 300 sample–SNP combinations (11.33%; Table S4). On average, we excluded 3.77 data points per SNP, with the total number of data points per SNP ranging from 32 to 35. The SNPs *fam178* and *supt6h_var1* showed the highest number of exclusions (six data points each), whereas *usp_var1* showed the fewest exclusions (one data point). Outliers corresponding to the *T. ivanbureschi* homozygote cluster accounted for 11.76% of all excluded data points, and *T. macedonicus* homozygotes accounted for 26.47%. F1 hybrid genotypes represented the majority of excluded outliers (61.76%), consistent with differential allele‐specific primer competition when both alleles are present. We estimated centroids using an average of 14.44 data points for heterozygotes and 7.61 data points for each homozygote, corresponding to 80.22% and 84.55% of the total animals included in the experiment, respectively. The angles obtained from homozygous centroids showed relatively narrow ranges across all SNPs: αA ( *T. ivanbureschi* homozygous) ranged from 80.8° to 84.2° (mean = 82.0°, SD = 1.13), and αB ( *T. macedonicus* homozygous) ranged from 10.6° to 15.2° (mean = 12.9°, SD = 1.46). These angles indicate slight deviations of the homozygous centroids from their theoretical positions (αA = 90°, αB = 0°), consistent with background fluorescence inherent to KASP assays. In contrast, αAB (F1 hybrid cluster) displayed a wider range, from 35.0° to 54.3° (mean = 46.6°, SD = 6.05), as expected due to variability in allele‐specific primer competition that our SNP‐level calibration is designed to absorb (centroid angles in, Table S5). Figure S1 shows KASP plots of animal swabs before treating outliers, and the clusters retained for the calculation of αA, αAB and αB.

Evaluation of SNP‐specific bias from *HI* estimates showed that all SNPs exhibited deviations below 1%, with combined confidence intervals within 5% of the *HI* scale; therefore, all SNPs were retained for statistical analyses (median SNP bias and confidence intervals in Figure S2).

Comparison of genotypes obtained through animal swabs versus eDNA samples revealed a strong Spearman's rank correlation between *HI*_obs_ and *HI*_*exp*_ in both sampling regimes (low conc. eDNA: ρ = 0.896, *p* < 0.0001; high conc. eDNA: ρ = 0.930, *p* < 0.0001). Permutation‐based ANCOVA showed no significant effect of sampling regime (*p* = 0.56) or of the interaction between *HI*_*exp*_ and sampling regime (*p* = 0.84), indicating that eDNA concentration did not alter the overall relationship between *HI*_obs_ and *HI*_*exp*_ (Table S6).

**FIGURE 2:**
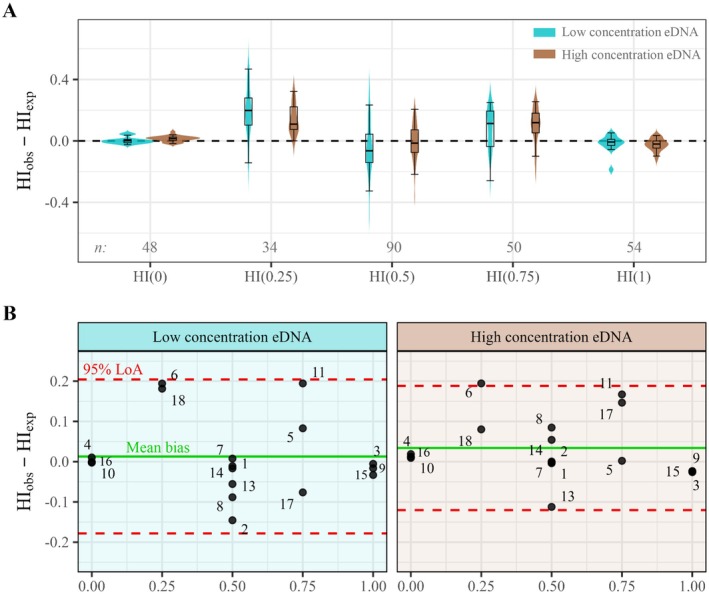
Accuracy of eDNA‐derived hybrid index (*HI*) estimates across genotypic profiles and sampling regimes. (A) Violin and box plots of deviations between observed *HI* from eDNA samples and expected *HI* from skin‐swab genotypes (*HI*_obs_—*HI*_exp_). Genotypic profiles (*HI* = 0, 0.25, 0.5, 0.75, 1) represent the expected *Triturus macedonicus* allele proportion derived from verified genotypes. Observed *HI* values correspond to each sample–SNP combination. n, along plot bottom, denotes the number of observed *HI* values per genotypic profile; half of n corresponds to each eDNA sampling regime. The dashed line marks zero bias. Positive values indicate bias towards *T. macedonicus* ; negative values towards *T. ivanbureschi* . Box centers show medians, boxes the interquartile range (IQR), and whiskers 1.5 × IQR. (B) Bland–Altman plots of population bias shown separately for each eDNA sampling regime. Each point represents one population whose *HI* estimates are averaged across SNPs. Positive values indicate a shift towards *T. macedonicus* ; negative values towards *T. ivanbureschi* . The green solid line shows the mean bias, and red dashed lines the 95% limits of agreement (LoA = mean ± 1.96 SD).

Assessing whether bias in *HI* estimation depended on genotypic profile, Kruskal–Wallis tests detected significant differences among profiles in both eDNA sampling regimes (low conc. eDNA: χ^2^ = 30.4, *p* < 0.0001; high conc. eDNA: χ^2^ = 47.82, *p* < 0.0001). In the low concentration sampling regime, Wilcoxon signed‐rank tests showed a marked bias toward amplification of *T. macedonicus* alleles at *HI* (0.25) (median bias = 0.198 *HI* units, *p*_adj_ = 0.0019) and a moderate bias in the same direction at *HI* (0.75) (median bias = 0.113 *HI* units, *p*_adj_ = 0.049). In the high concentration sampling regime, *HI* (0.25) (median bias = 0.109 *HI* units, *p*_adj_ < 0.0001) and *HI* (0.75) (median bias = 0.118 *HI* units, *p*_adj_ = 0.001) also showed moderate bias towards *T. macedonicus* . Additionally, in the high concentration sampling regime, *HI* (0) showed a very small bias toward *T. macedonicus* (median bias = 0.015 *HI* units, *p*_adj_ = 0.005), whereas *HI* (1) showed a very small bias towards *T. ivanbureschi* (median bias = −0.02 *HI* units, *p*_adj_ < 0.0001) (Figure 2A). Pairwise variance comparisons using the Fligner–Killeen test indicated a larger variance for heterozygous than for homozygous profiles in both sampling regimes (all comparisons significant after adjustment; *p*_adj_ ≤ 0.0003).

Evaluation of sampling regime‐specific accuracy and precision using Bland–Altman analysis showed a slightly lower mean bias (i.e., higher accuracy) at low than at high eDNA concentration (0.0129 at low conc. vs. 0.0341 at high conc.). However, the limits of agreement were 19.4% narrower at high concentration (−0.178 to 0.204 at low conc. vs. −0.120 to 0.188 at high conc.), corresponding to a 35% reduction in variance and indicating greater precision under high eDNA concentration (Figure 2B). Analysis of differences in *HI* estimates between sampling regimes yielded a mean difference of 0.021 *HI* units with 95% LoA of −0.191 to 0.148, which reflects moderate variability but high reproducibility on average across both eDNA concentrations (Figure S3).

Biomass correction in mixed‐genotype populations with unequal individual body weights did not alter *HI* estimates (paired Wilcoxon signed‐rank tests yielded median differences of −0.012 to 0.014 *HI* units, with *p*_adj_ 0.57–1). Thus, individual body weight did not significantly influence bias (weight of the animals in, Table S7).

## 4 Discussion

Our objective was to develop an eDNA‐based approach to screen for populations of mixed ancestry by amplifying species‐specific nuclear SNPs and calculating a hybrid index from end‐point KASP fluorescence. We selected as a model system a species pair for which prior evidence indicates permeable reproductive barriers; consequently, these species do not remain strictly syntopic but hybridise upon contact. In this context, the simultaneous detection of fluorescence from diagnostic alleles of both species is indicative of a hybrid population. We found strong concordance between the expected ancestry derived from individual skin swabs and the observed ancestry estimates obtained from eDNA collections, providing high confidence in ancestry inference at the population scale. The correlation remained consistently strong across the two mesocosm eDNA concentrations, with no detectable effect of concentration, indicating that both sampling regimes yielded highly similar estimates. Although the high concentration sampling regime (≈5 × eDNA) reduced the variance of hybrid index estimation by 35%, the low concentration sampling regime differed in mean bias by only ~2%. These results indicate that ancestry estimates are robust and informative under various eDNA sampling conditions.

Here, we demonstrate that hybrid populations can be screened from nuclear eDNA without sequencing, which represents a foundational step for hybridisation research and a practical advance for eDNA applications in evolutionary ecology (Stewart and Taylor 2020). However, our approach cannot identify hybrid individuals and therefore cannot replace individual sampling. As such, it cannot resolve the proportions of F1s, F2s and successive backcrosses. The ancestry estimates derived from eDNA instead represent a pooled signal of all detectable individual sequences within a population. This is conceptually equivalent to pooling DNA extracts from multiple individuals (Pool‐Seq) and then mapping allele‐frequency changes as geographic clines across hybrid zones (e.g., Rafati et al. 2018). Compared with Pool‐Seq, however, the eDNA‐based approach is substantially more cost and time efficient, as Pool‐Seq requires the capture and handling of organisms, individual sampling and separate DNA extractions for each specimen. By contrast, eDNA sampling requires only a single DNA extraction from an environmental sample to capture the mixture of individual sequences needed for ancestry inference (Sigsgaard et al. 2020), thereby eliminating the need to plan capture logistics and obtain collection permits. Moreover, integrating eDNA sampling with KASP genotyping further reduces costs, as this technique is affordable for small SNP panels (Wielstra et al. 2016) and does not require sequencing. Collectively, these advantages establish our approach as a non‐invasive, rapid and cost‐efficient screening tool for prospecting hybridisation.

Using eDNA to sieve putatively hybrid populations from pure populations could allow rapid screening to identify where hybridisation is potentially most pervasive, and it may also yield valuable insights into the detailed analysis of hybrid zone characteristics. Similar to Pool‐Seq, eDNA could potentially allow for inferences through clinal analysis. For instance, eDNA‐derived ancestry might be used to infer the center of a hybrid zone (where *HI*≈0.5), the strength of selection against hybrids (reflected in the steepness of SNP‐specific clines), the width of the hybrid zone (clinal width; 1/slope at the cline center), and whether the hybrid zone is moving and in which direction (SNP‐specific cline displacement and asymmetric tails of SNPs) (Barton and Gale 1993; Wielstra 2019). Nevertheless, a critical distinction must be made between the ‘environmental pooling’ inherent to eDNA and the ‘laboratory pooling’ of Pool‐Seq. While Pool‐Seq allows DNA input to be equalised per individual and population size is known, eDNA pools form naturally, and are thus subject to biological sources of variance, with population size inferred indirectly through mixture models (Andres et al. 2021). In small populations, for example, factors such as unequal DNA shedding or spatial heterogeneity in eDNA distribution may disproportionately shift observed allele ratios (Klymus et al. 2015; Thalinger et al. 2021; Troth et al. 2021). Conversely, in large populations, the eDNA signal may benefit from an ‘averaging effect’ that smooths out stochastic variation and unequal shedding (Didaskalou et al. 2026). However, this aggregate signal could also become less sensitive to rare alleles if their genetic contribution is masked by the dominant allele (Adams et al. 2023; Andres et al. 2023).

These sources of variation might be at least partially mitigated by increasing the volume of water filtered, as evidenced by the reduction in variance observed in our high concentration sampling regime compared with the low concentration one. Additionally, unlike our controlled experiment where we sampled water from containers housing only two animals for 24 h, natural systems may allow for higher detectability as eDNA accumulates over time (Jo et al. 2020). Consequently, although ancestry estimates derived from eDNA should be interpreted cautiously and ideally validated with individual‐level data, our mesocosm results provide a robust baseline for screening hybrid populations. This approach remains a valuable screening tool that could be particularly useful for improving the efficiency of conventional monitoring by flagging sites where hybridisation is most likely to occur, thereby focusing individual sampling efforts on locations of greatest relevance.

While we selected a model system with known permeable reproductive barriers, evaluating (in)concordance among SNP ancestry estimates within populations could, in principle, also help distinguish syntopy from hybrid populations in systems where prior evidence of hybridisation is lacking (Barton and Hewitt 1985; Rieseberg et al. 1996; Buggs 2007; Stewart and Taylor 2020). Although our eDNA toolkit proved effective for ancestry estimation in mesocosms, the present experimental design does not allow us to empirically differentiate between syntopic species and hybrid populations, because the mesocosms contained only pure individuals and/or F1 hybrids. Since all SNPs used are species‐diagnostic, populations composed of F1 hybrids showed *HI≈0.5* across all loci, and the same pattern would arise from a 1:1 mixture of the two parental species without hybridisation; however, this scenario is precluded in our model system due to the permeability of reproductive barriers. Distinguishing syntopic species from a hybrid population when the strength of reproductive barriers is unknown in a practical field setting is theoretically possible, because hybrid zones are typically characterised by the presence of introgressed individuals produced by multiple backcrosses (Anderson and Hubricht 1938; Harrison and Larson 2014). These individuals exhibit locus‐by‐locus discordance (Rieseberg et al. 1996), a pattern that is particularly evident when using unlinked markers that segregate independently, as in this study (Arntzen et al. 2018). This locus‐by‐locus discordance results in heterogeneous genomic permeability, whereby specific barrier loci resist gene flow (maintaining ancestry values near parental extremes; *HI≈*0 or 1), while others permissive or adaptive attain elevated frequencies or even fixation. Simultaneously, the frequencies of neutral loci are shaped by the stochasticity of recombination and the cumulative history of backcrossing. This complex mosaic of allele frequencies, especially when repeatable across replicates or along multiple transects, signals introgression and is fundamentally inconsistent with mere syntopy (which would yield the same allele frequency at all loci). At present, generating introgressed individuals in captivity for our study taxa remains challenging because these newts mature slowly, and preliminary results (Vučić et al. 2022) suggest reduced survival beyond early hybrid generations. It may therefore be preferable to focus on field surveys. Consequently, while this proof‐of‐concept offers immediate utility for systems lacking strong reproductive barriers, assessing introgression signatures in natural populations is the necessary next step.

Despite the generally good performance of our toolkit, the accuracy of ancestry estimates varied with the genotypic profile of the populations. Homozygous samples were detected accurately with low variance, consistent with previous eDNA studies using KASP (Schabacker et al. 2020; van Kuijk et al. 2025). In heterozygous populations, variance was greater than in homozygous populations, but this did not necessarily introduce bias into the estimates. The *HI* (0.5) genotypic profile, where both alleles were equally represented, showed high accuracy with no detectable bias. In contrast, the two profiles with unequal allele proportions (*HI* (0.25) and *HI* (0.75)) exhibited a moderate bias towards the *T. macedonicus* diagnostic alleles, with median deviations of ~10%–20%. This bias may arise from biological factors. For example, some individuals may have contributed more to the eDNA pool than others (Klymus et al. 2015; Thalinger et al. 2021), disproportionately shifting allele ratios due to unequal shedding (cf. Andres et al. 2021). Nevertheless, biomass correction did not reduce bias for *HI* (0.25) and *HI* (0.75), indicating that any unequal contribution, if present, was not related to differences in individual body weight. Additionally, since these populations consisted exclusively of male individuals, potential sex‐specific contribution differences can also be ruled out as a factor for the observed deviations.

Technical factors such as insufficient replication and non‐specific amplification may also have contributed to the observed deviations. Owing to experimental simplification and the exclusion of several mismatched genotypes, our biological replication design was unbalanced (six replicates at *HI* (0.5), three at *HI* (0.75), two at *HI* (0.25)), with the increased replication at *HI* (0.5) demonstrating the lowest deviations. Additionally, technical replication may have been insufficient: we performed four KASP reactions per sample–SNP combination, whereas Veldhoen et al. (2016) showed that detection error in low‐abundance eDNA targets can be greatly reduced by increasing replicates. Within the animal validation dataset, apart from the mislabeled individual M12A, 95.24% of the observed genotypes matched expectations. Although the remaining calls were unexpected and may reflect true genotypes, they may also represent false negatives and positives. In 11 of these 15 cases (3.49%), expected F1 hybrids produced homozygous signals, suggesting false negatives consistent with allelic dropout, whereby one of the two alleles fails to amplify. This pattern would be consistent with KASP genotyping results reported by Wielstra et al. (2016) for animal tissue samples and may be even more pronounced in eDNA samples where DNA concentrations are typically lower. The remaining four mismatches corresponded to expected homozygotes appearing heterozygous. These potential false positives for one of the two alleles accounted for 1.27% of the validation dataset, a proportion comparable to that reported by Wielstra et al. (2016; 2.1%). In the eDNA samples, neither false negatives/positives nor a signal detected in one field negative control appeared to substantially affect inference, given the high concordance between observed and expected values. However, in natural populations (especially where individual‐based validation is not feasible), future studies should substantially increase both biological and technical replication, add multiple negative and positive controls and rigorously implement contamination‐control procedures.

To our knowledge, this is the first empirical validation of the use of nuclear eDNA to screen for hybridisation at the population level. Our approach provides a non‐invasive and efficient means of identifying populations with mixed ancestry, making it potentially well suited for screening and tracking hybrid zones as a complement to individual‐based sampling. A practical workflow for future research would be to flag sites with intermediate ancestry and discordance across loci (a hallmark of hybridisation rather than syntopy) and then deploy individual‐based assays at those locations to quantify hybrid classes. Our next step will be direct field trials to test whether nuclear eDNA can be used to estimate ancestry in natural populations. If successful, this toolkit could enable early detection of hybrid zones and support more timely responses to the biodiversity crisis driven by human‐mediated hybridisation.

## Author Contributions

**Daniel Zumel:** formal analysis, methodology, visualisation, writing – original draft preparation, writing – review and editing. **Emilie Didaskalou:** investigation, methodology, writing – review and editing. **Tijana Vučić:** methodology, resources, writing – review and editing. **Milena Cvijanović:** methodology, resources, writing – review and editing. **Ana Ivanović:** methodology, resources, writing – review and editing. **Maja Ajduković:** methodology, resources, writing – review and editing. **Ben Wielstra:** conceptualisation, investigation, methodology, supervision, writing – review and editing. **Anagnostis Theodoropoulos:** methodology, writing – review and editing. **Kathryn Stewart:** conceptualisation, funding acquisition, investigation, methodology, project administration, supervision, writing – review and editing.

## Funding

This work was supported by Nederlandse Organisatie voor Wetenschappelijk Onderzoek, VI.Vidi.213.088, Serbian Ministry of Science, Technological Development and Innovation, 451‐03‐136/2025‐03/200007, 451‐03‐136/2025‐03/200178 and 451‐03‐137/2025‐03/200178.

## Disclosure

Benefit‐Sharing Statement: This research complied with national laws implementing the Convention on Biological Diversity and the Nagoya Protocol, under collection permits from Serbia and Montenegro and institutional ethics approval. Benefits derive from open dissemination of results and materials, and from conservation applications as described in the manuscript.

## Ethics Statement

Permits for collecting individuals for the experiment were granted by the Serbian Ministry of Energy, Development and Environmental Protection (permit No. 353‐01‐75/2014‐08), Serbian Ministry for Environmental Protection (permit No. 353‐01‐1506/2022‐04) and the Agency for Environmental Protection, Montenegro (permit No. UPI‐328/04). The experimental procedures were approved by the Ethics Committee of the Institute for Biological Research ‘Siniša Stanković,’ University of Belgrade (decision no. 323–07‐07479/2023–05). All experimental animals were treated in compliance with the European directive (2010/63/EU) on the protection of animals used for experimental and other scientific purposes.

## Conflicts of Interest

The authors declare no conflicts of interest.

## Supporting information


**Figure S1:** KASP fluorescence scatterplots of samples from animal swabs used for the validation of environmental samples. Each page shows one SNP. Left: Genotype validation data prior to the removal of outliers and controls. Right: data retained for estimating centroid angles for each genotype. Points are coloured by expected genotype ( *T. ivanbureschi* in black, F1 Hybrid in grey, *T. macedonicus* in white). Negative controls are shown in blue. Yellow hollow circles denote samples flagged as ‘Mismatched genotype’ when their fluorescence signal was closer to a different genotype centroid than to the expected one. Labelled animal samples not flagged in yellow correspond to the outliers excluded for the calculation of centroid angles (Euclidean distance ≥ median + 3 × MAD). Axes show FAM (x) and HEX (y) fluorescence; plot limits are fixed to [0,4] for comparability across SNPs.

**Figure S2:** SNP bias in Hybrid Index (HI) with bootstrap uncertainty. For each SNP, the point shows the median bias estimated from samples taken from skin swabs with confirmed genotypes; horizontal bars indicate 95% bootstrap confidence intervals for the median (B = 2000 resamples). The dashed vertical line marks zero bias. SNPs were retained for downstream analyses when their entire 95% CI lay within ±0.05.

**Figure S3:** Paired Bland–Altman comparing estimates from each sampling regime. Each point is a population sampled under both regimes, whose HI estimates are averaged across SNPs. Positive values indicate that low concentration (conc.) eDNA sampling yields higher HI than high conc. eDNA sampling for the same population; negative values indicate the opposite. Green solid line is the mean bias and red dashed lines the 95% limits of agreement (LoA = mean ±1.96 SD). Mean bias was 0.021 with LoA [−0.191, 0.148], indicating minimal average discrepancy between sampling regimes.

**Table S1:** SNPs targeted. The first variant, before the /, is diagnostic for *Triturus macedonicus* , while the second, after the /, is diagnostic for *T. ivanbureschi* . Data from Arntzen et al. (2018). Full reference: Arntzen, J. W., Üzüm, N., Ajduković, M. D., Ivanović, A., & Wielstra, B. (2018). Absence of heterosis in hybrid crested newts. PeerJ, 6, e5317.

**Table S2:** KASP reactions performed. Each row represents a PCR and includes raw fluorescence measurements for FAM and HEX channels, SNP name, sample identifier (SubjectID), dataset type, expected value of hybrid index (HI_exp), and associated experimental metadata.

**Table S3:** Mismatched genotypes found in the individual skin swab samples used for validating eDNA data. Each row corresponds to an animal–SNP combination whose coordinates fell closer to the centroid of a genotype other than the one assigned based on sample metadata; these genotypes were excluded from downstream analyses, and the associated environmental samples were removed for the corresponding SNPs. Columns: SNPID, SNP name, SubjectID, individual identifier; assigned_Genotype, genotype inferred from species identity based on collection records; nearest_genotype, genotype of the closest centroid in the FAM–HEX plane; FAM and HEX, fluorescence intensities after replicate averaging; own_dist, Euclidean distance to the assigned‐genotype centroid; nearest_dist, Euclidean distance to the nearest centroid; eDNA_associated, IDs of eDNA samples excluded alongside the mismatched call.

**Table S4:** Outlier genotypes excluded from the calculation of centroid angles. Each row corresponds to an individual–SNP combination whose fluorescence centroid (FAM, HEX) fell beyond the median + 3 × MAD Euclidean‐distance threshold from the centroid of its assigned‐genotype cluster. Columns: SNPID, SNP name; SubjectID, animal identifier; Assigned_Genotype, genotype inferred from species identity based on collection records; FAM and HEX, fluorescence intensities after replicate averaging; Genotype_FAM and Genotype_HEX, fluorescence coordinates of the assigned‐genotype centroid; dist_to_genotype, Euclidean distance from the subject centroid to its assigned‐genotype centroid; threshold, distance cutoff (median + 3 × MAD) for the corresponding SNP–genotype cluster. Values exceeding the threshold were flagged as outliers, removed from centroid estimation, and excluded from the calculation of centroid angles. Outliers flagged as mismatched genotypes are shown in Table S3.

**Table S5:** Angular positions of genotype centroids used to map fluorescence ratios to the hybrid index (*HI*) scale. For each SNP, centroid angles (in degrees) are calculated from the origin of the FAM–HEX fluorescence plane, with 0° aligned to the FAM axis and 90° to the HEX axis. alpha_A: centroid angle for *T. ivanbureschi* homozygotes (*HI* = 0); alpha_AB: centroid angle for F1 hybrids (*HI* = 0.5); alpha_B: centroid angle for *T. macedonicus* homozygotes (*HI* = 1). These angles were derived after low‐fluorescence filtering, averaging across PCR replicates, and outlier removal.

**Table S6:** Observed Hybrid Index (*HI_obs*) for all sample–SNP combinations. Columns: SNPID, SNP name; SubjectID, sample name; Dataset, origin of the data (skin swabs or eDNA); alpha sample, angle derived from FAM and HEX fluorescence readings; Sampling, eDNA sampling regime (NA for skin swabs); Genotypic profile, expected hybrid index based on verified allele composition (NA for skin swabs); *HI_exp*, expected HI for all samples (eDNA and skin swabs); *HI_obs*, observed *HI*.

**Table S7:** Individual body weights of animals from mixed‐genotype populations used to assess the effect of biomass correction on hybrid index estimates (HI). Columns: SubjectID_animal, identifier for each animal; SubjectID_population, identifier of the population to which the animal belongs; Weight, individual body weight (g); HI_exp_animal, individual expected HI value; HI_exp_population, expected HI value for the population (uncorrected for biomass).

## Data Availability

The data that support the findings of this study are available in the article and its Supporting Information. The code used to perform the analyses is publicly available on Zenodo at https://doi.org/10.5281/zenodo.17332357.
