# Supplementary material for: Hybrid Horizons: Screening Hybridisation Through Nuclear Environmental DNA
Source: Mol Ecol Resour. 2026 May 4;26:e70134. doi: 10.1111/1755-0998.70134 (PMC13137079; doi:10.1111/1755-0998.70134)
Supplement: Supplementary file 1 — Figure S1: KASP fluorescence scatterplots of samples from animal swabs used for the validation of environmental samples. Each page shows one SNP. Left: Genotype validation data prior to the removal of outliers and controls. Right: data retained for estimating centroid angles for each genotype. Points are coloured by expected genotype ( T. ivanbureschi in black, F1 Hybrid in grey, T. macedonicus in white). Negative controls are shown in blue. Yellow hollow circles denote samples flagged as ‘Mismatched genotype’ when their fluorescence signal was closer to a different genotype centroid than to the expected one. Labelled animal samples not flagged in yellow correspond to the outliers excluded for the calculation of centroid angles (Euclidean distance ≥ median + 3 × MAD). Axes show FAM (x) and HEX (y) fluorescence; plot limits are fixed to [0,4] for comparability across SNPs. [file MEN-26-e70134-s003.pdf]

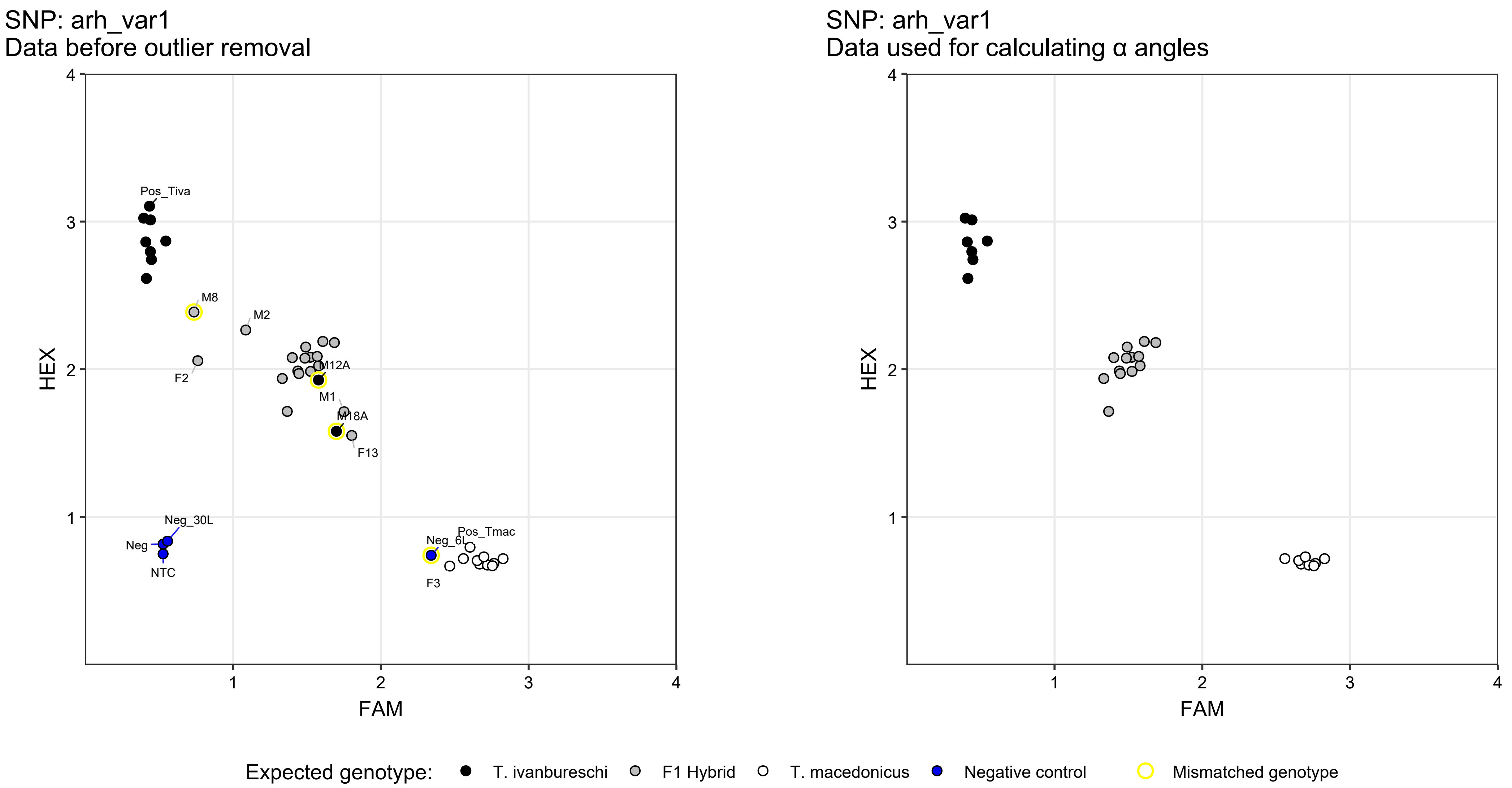

SNP: arh_var1
Data before outlier removal
SNP: arh_var1
Data used for calculating α angles
HEX
FAM
Pos_Tiva
M8
M2
F2
M12A
M1
M18A
F13
Neg_30L
Neg
NTC
Neg_6L
Pos_Tmac
F3
Expected genotype:
T. ivanbureschi
F1 Hybrid
T. macedonicus
Negative control
Mismatched genotype

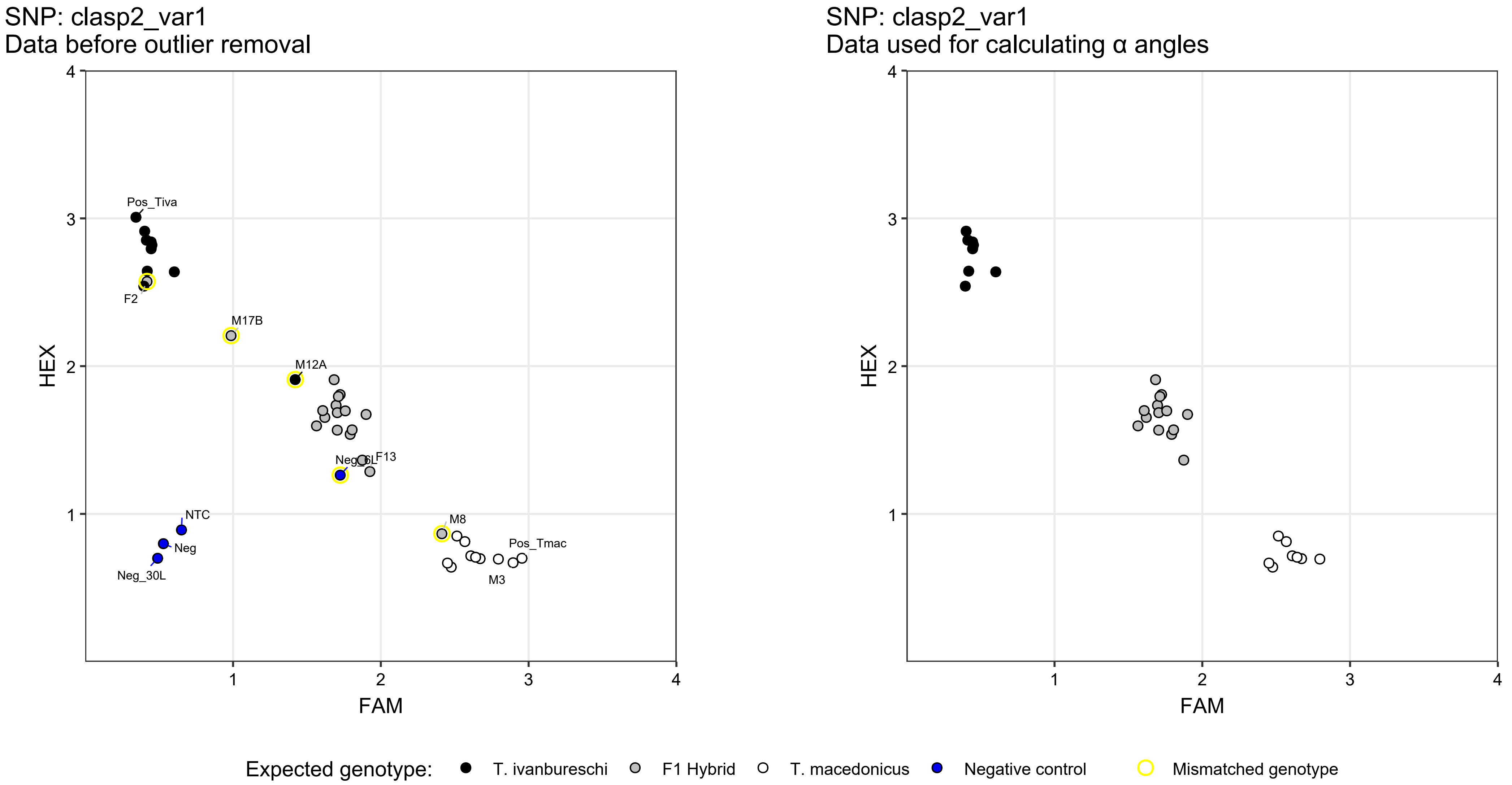

SNP: clasp2_var1
Data before outlier removal
SNP: clasp2_var1
Data used for calculating α angles
HEX
FAM
Pos_Tiva
F2
M17B
M12A
Neg_6L
F13
NTC
Neg
Neg_30L
M8
Pos_Tmac
M3
Expected genotype:
T. ivanbureschi
F1 Hybrid
T. macedonicus
Negative control
Mismatched genotype

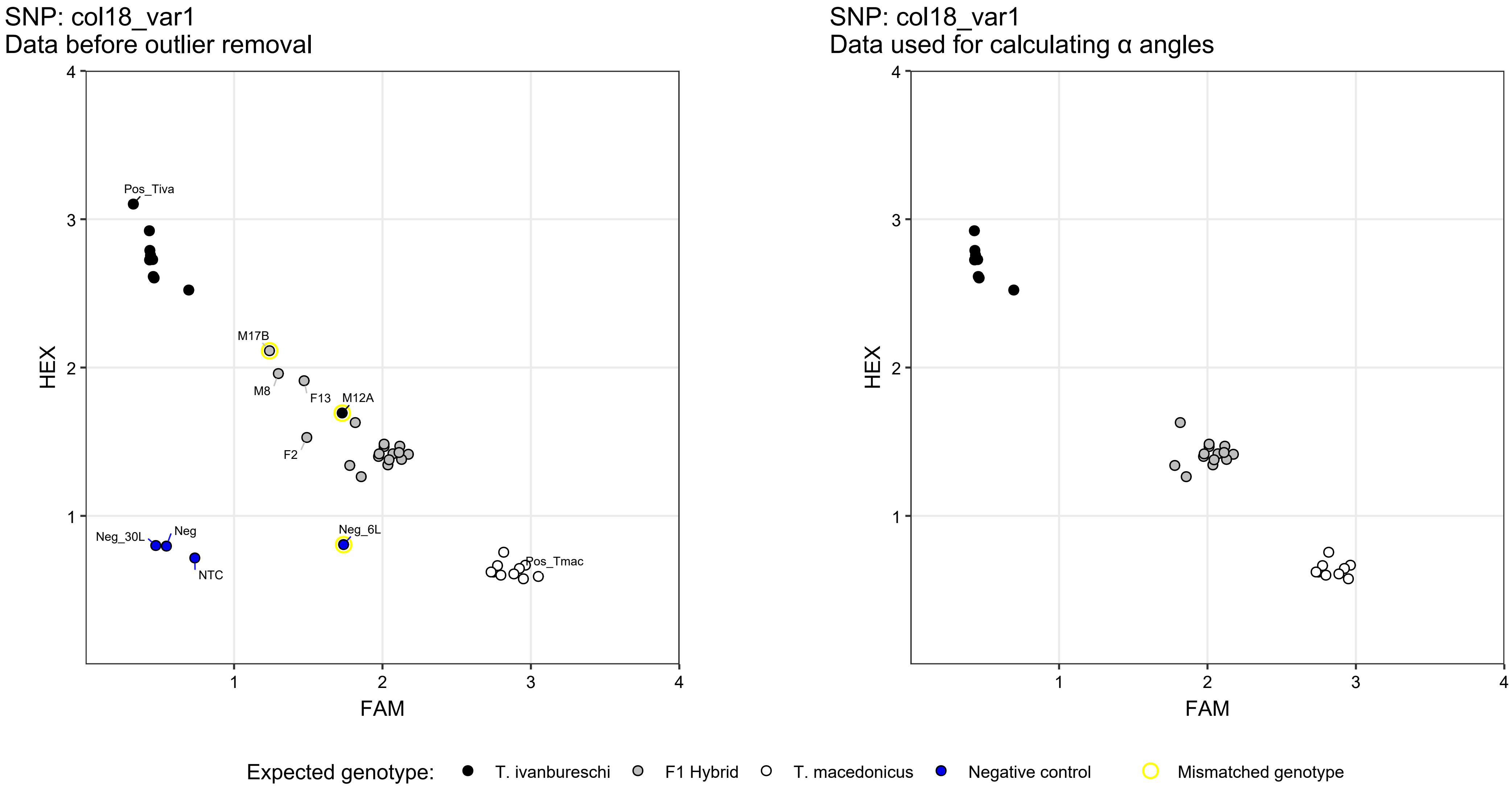

SNP: col18_var1
Data before outlier removal
SNP: col18_var1
Data used for calculating α angles
HEX
FAM
Pos_Tiva
M17B
M8
F13
M12A
F2
Neg_30L
Neg
NTC
Neg_6L
Pos_Tmac
Expected genotype:
T. ivanbureschi
F1 Hybrid
T. macedonicus
Negative control
Mismatched genotype

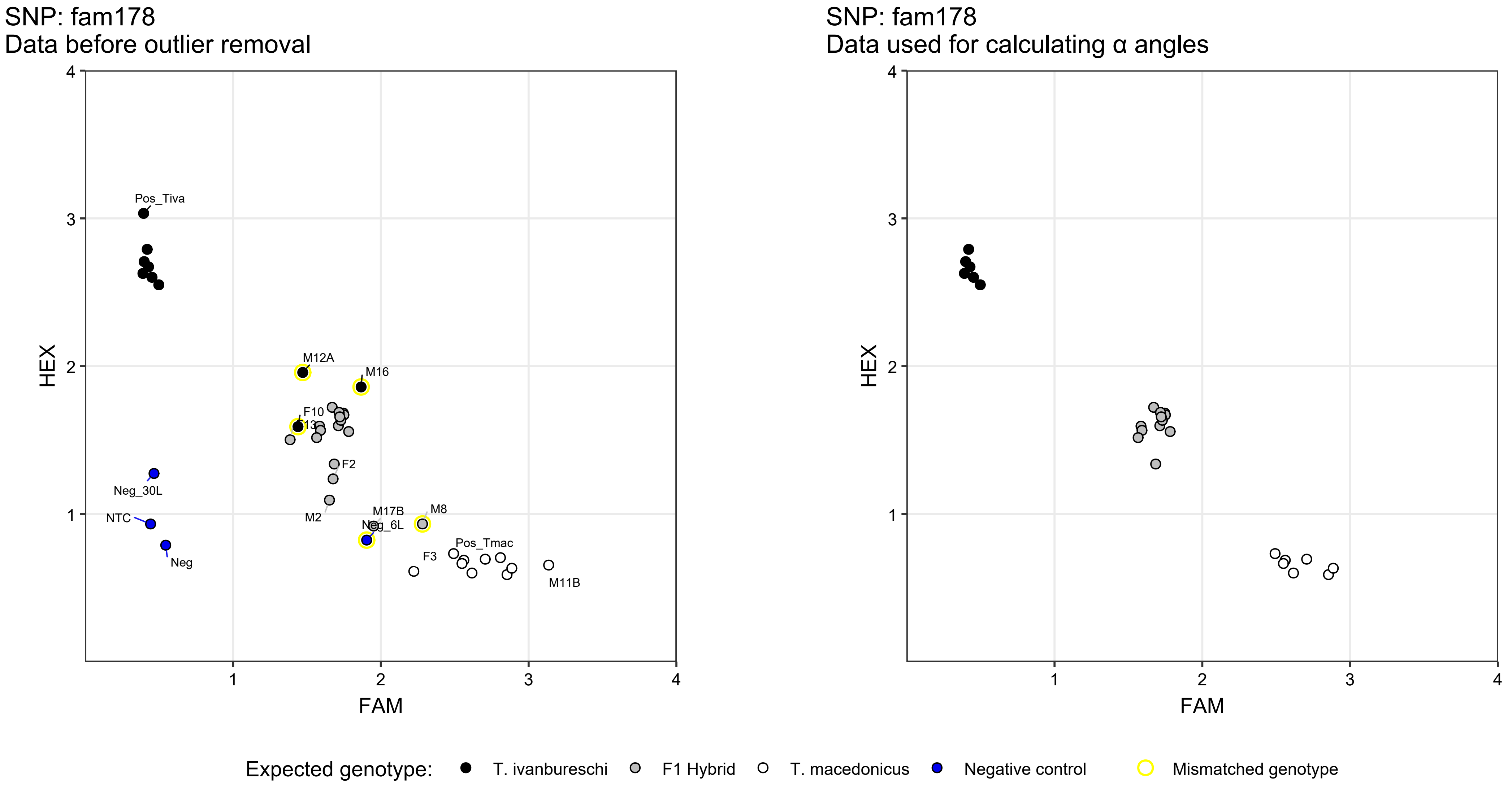

SNP: fam178
Data before outlier removal
SNP: fam178
Data used for calculating α angles
HEX
FAM
Pos_Tiva
M12A
M16
F10
F13
F2
Neg_30L
NTC
Neg
M2
M17B
Neg_6L
M8
Pos_Tmac
F3
M11B
Expected genotype:
T. ivanbureschi
F1 Hybrid
T. macedonicus
Negative control
Mismatched genotype

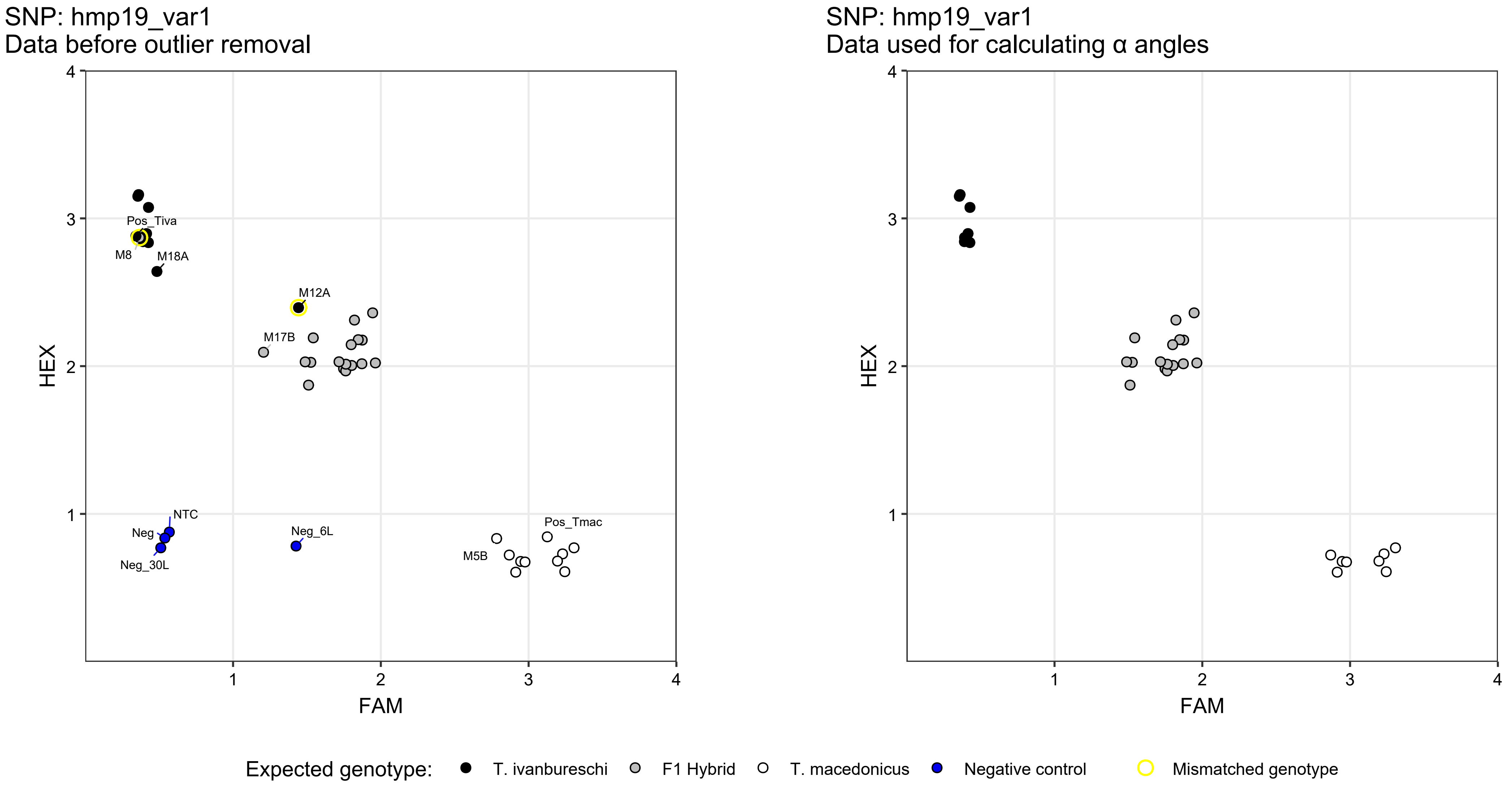

SNP: hmp19_var1
Data before outlier removal
SNP: hmp19_var1
Data used for calculating α angles
HEX
FAM
Pos_Tiva
M8
M18A
M12A
M17B
NTC
Neg
Neg_30L
Neg_6L
Pos_Tmac
M5B
Expected genotype:
T. ivanbureschi
F1 Hybrid
T. macedonicus
Negative control
Mismatched genotype

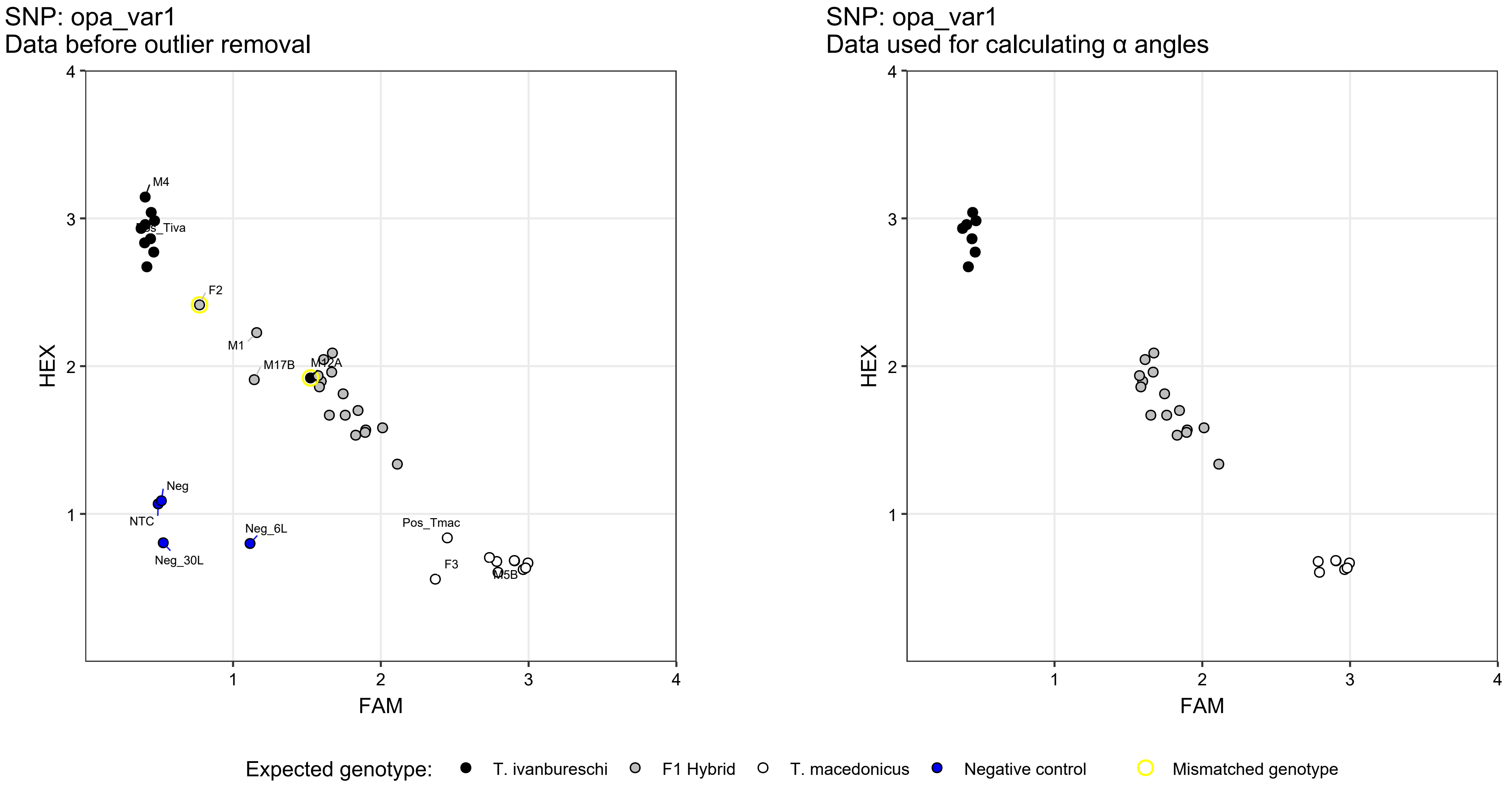
SNP: opa_var1
Data before outlier removal
SNP: opa_var1
Data used for calculating α angles
HEX
FAM
M4
Pos_Tiva
F2
M1
M17B
M12A
Neg
NTC
Neg_30L
Neg_6L
Pos_Tmac
F3
M5B
Expected genotype:
T. ivanbureschi
F1 Hybrid
T. macedonicus
Negative control
Mismatched genotype

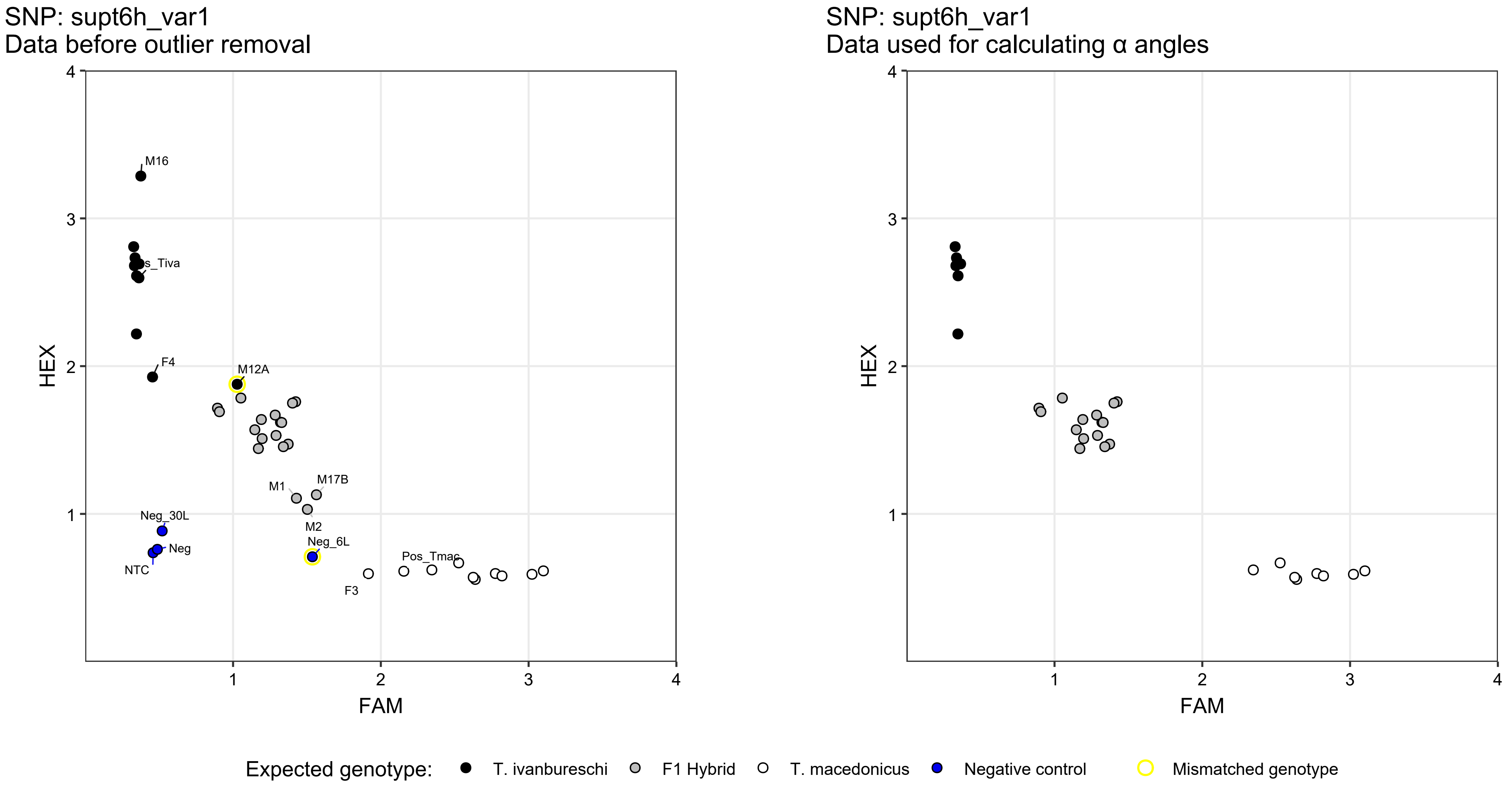

SNP: supt6h_var1
Data before outlier removal
SNP: supt6h_var1
Data used for calculating α angles
HEX
FAM
M16
Pos_Tiva
F4
M12A
M1
M17B
M2
Neg_30L
Neg
NTC
Neg_6L
Pos_Tmac
F3
Expected genotype:
T. ivanbureschi
F1 Hybrid
T. macedonicus
Negative control
Mismatched genotype

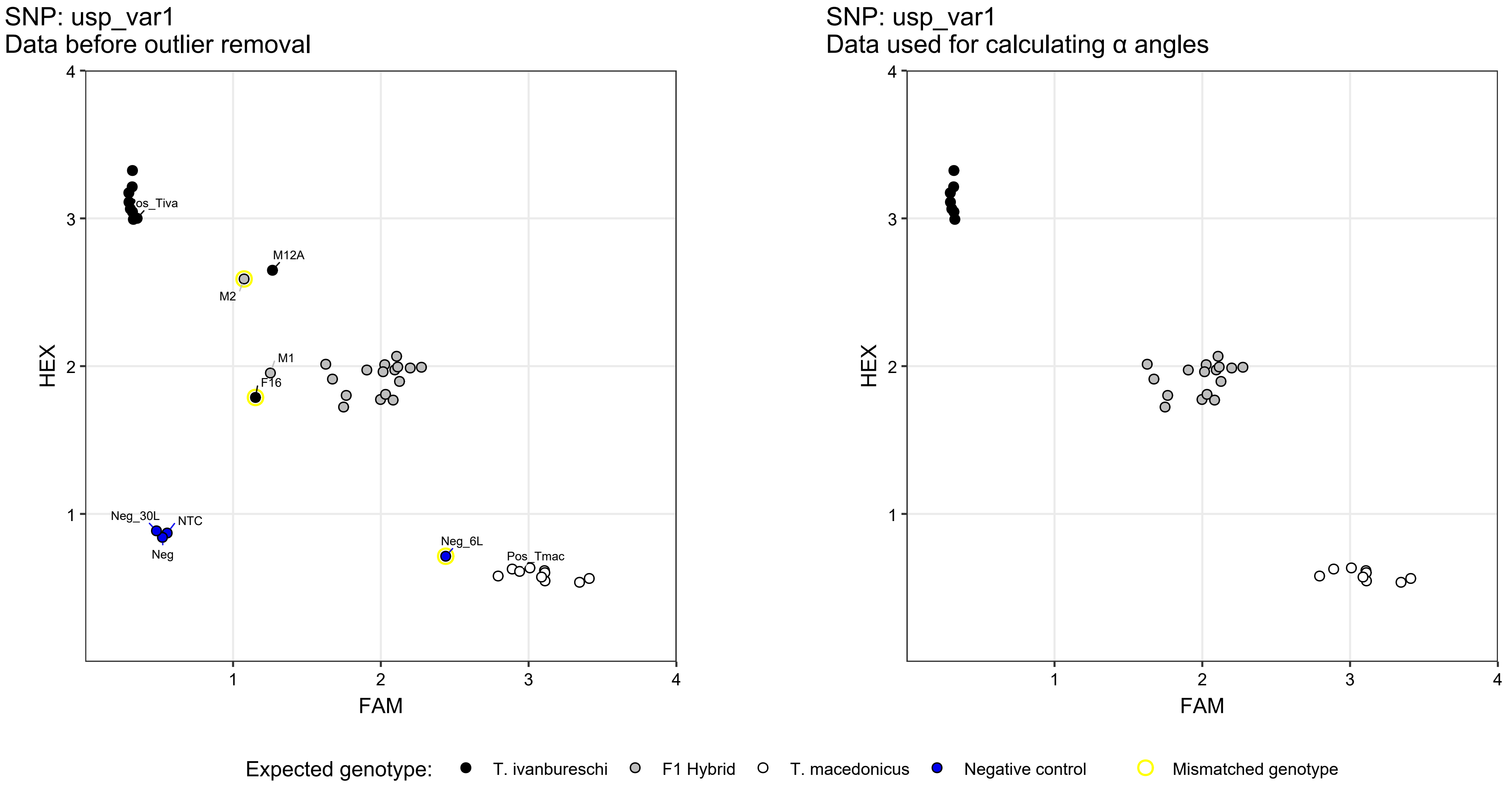
SNP: usp_var1
Data before outlier removal
SNP: usp_var1
Data used for calculating α angles
HEX
FAM
Pos_Tiva
M12A
M2
M1
F16
Neg_30L
NTC
Neg
Neg_6L
Pos_Tmac
Expected genotype:
T. ivanbureschi
F1 Hybrid
T. macedonicus
Negative control
Mismatched genotype

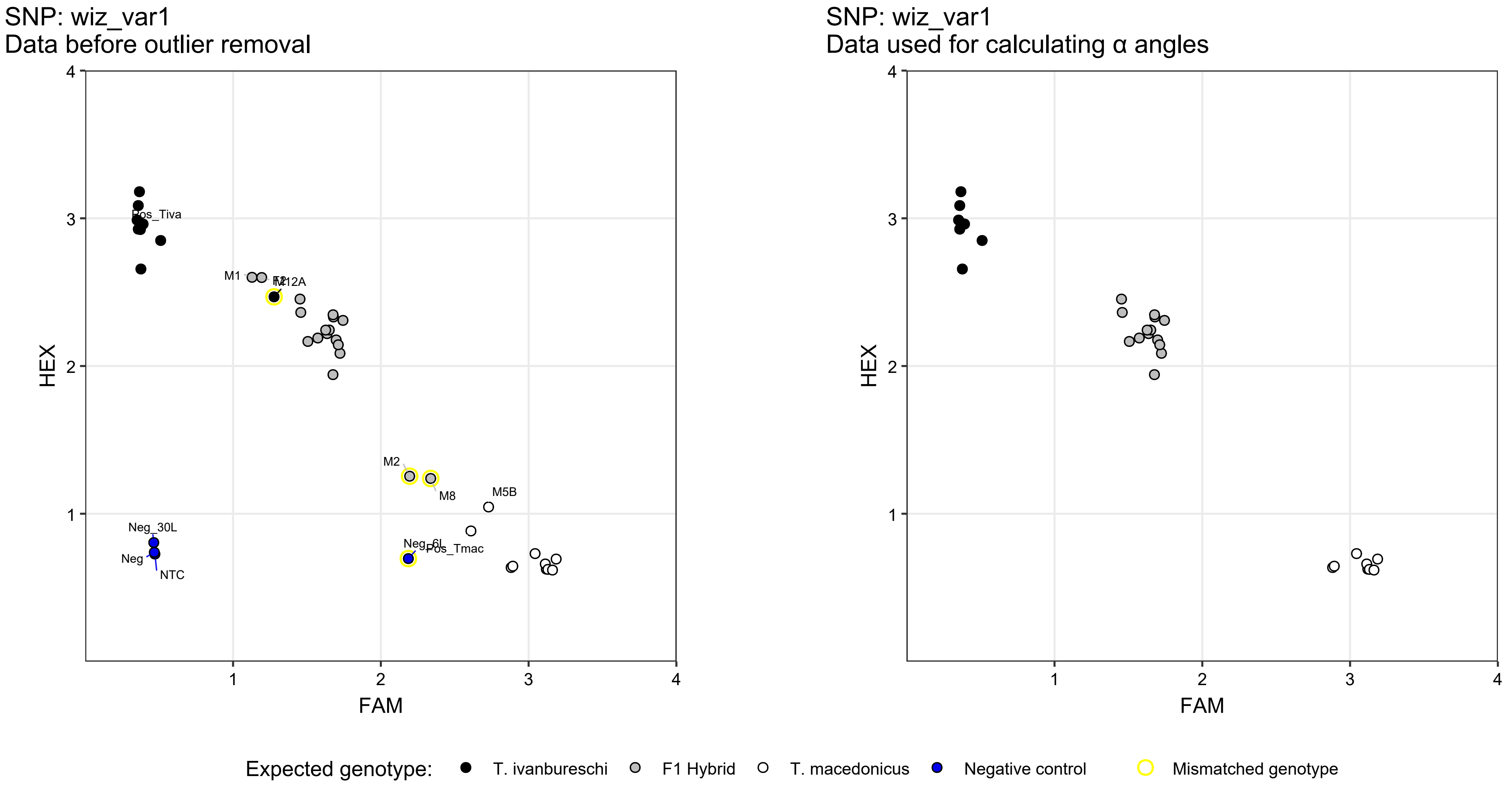
SNP: wiz_var1
Data before outlier removal
SNP: wiz_var1
Data used for calculating α angles
HEX
FAM
Pos_Tiva
M1
F2
M12A
M2
M8
M5B
Neg_30L
Neg
NTC
Neg_6L
Pos_Tmac
Expected genotype:
T. ivanbureschi
F1 Hybrid
T. macedonicus
Negative control
Mismatched genotype

Figure S1. KASP fluorescence scatterplots of samples from animal swabs used for the validation of environmetal samples. Each page shows one SNP. Left: Genotype validation data prior to the removal of outliers and controls. Right: data retained for estimating centroid angles for each genotype. Points are colored by expected genotype (*T. ivanbureschi* in black, F1 Hybrid in gray, *T. macedonicus* in white). Negative controls are shown in blue. Yellow hollow circles denote samples flagged as 'Mismatched genotype' when their fluorescence signal was closer to a different genotype centroid than to the expected one. Labelled animal samples not flagged in yellow correspond to the outliers excluded for the calculation of centroid angles (Euclidean distance ≥ median + 3×MAD). Axes show FAM (x) and HEX (y) fluorescence; plot limits are fixed to [0,4] for comparability across SNPs.
