# Supplementary material for: Hybrid Horizons: Screening Hybridisation Through Nuclear Environmental DNA
Source: Mol Ecol Resour. 2026 May 4;26:e70134. doi: 10.1111/1755-0998.70134 (PMC13137079; doi:10.1111/1755-0998.70134)
Supplement: Supplementary file 2 — Figure S2: SNP bias in Hybrid Index (HI) with bootstrap uncertainty. For each SNP, the point shows the median bias estimated from samples taken from skin swabs with confirmed genotypes; horizontal bars indicate 95% bootstrap confidence intervals for the median (B = 2000 resamples). The dashed vertical line marks zero bias. SNPs were retained for downstream analyses when their entire 95% CI lay within ±0.05. [file MEN-26-e70134-s007.pdf]

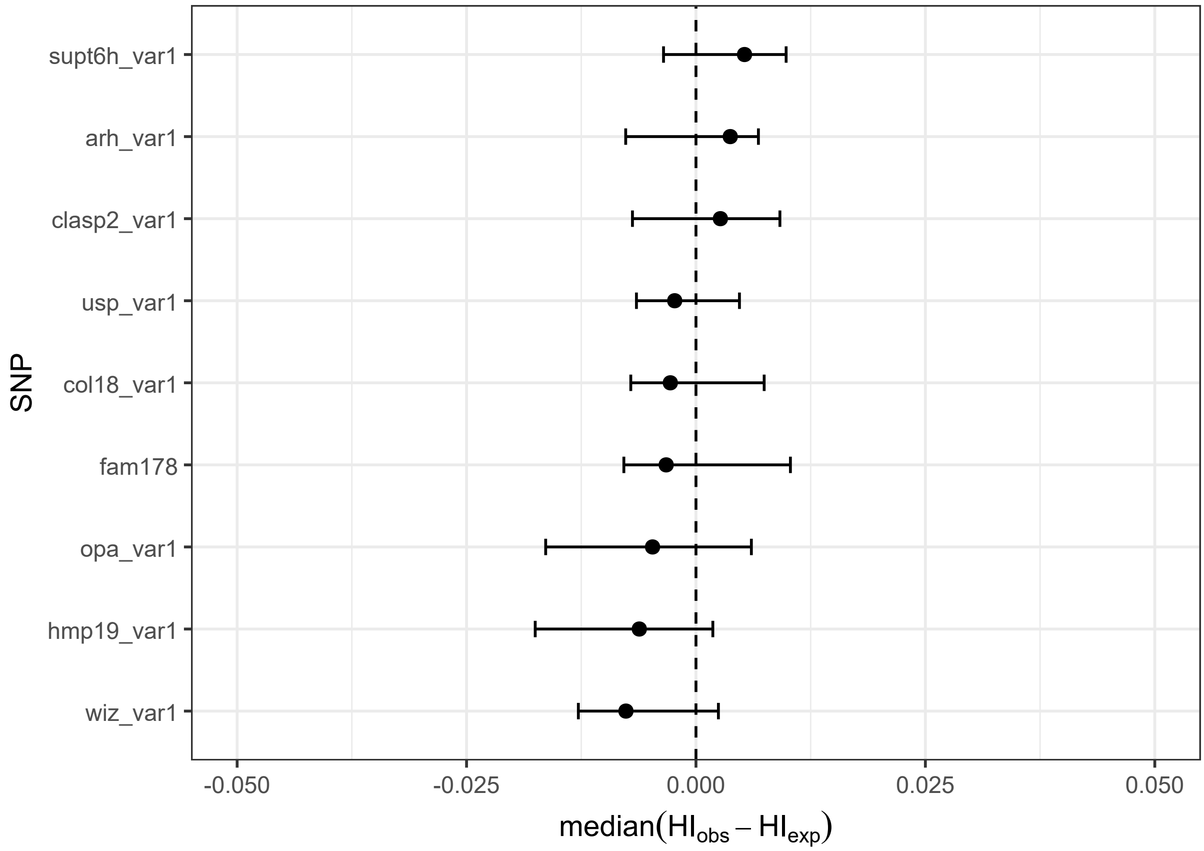

Figure S2. SNP bias in Hybrid Index (HI) with bootstrap uncertainty. For each SNP, the point shows the median bias estimated from samples taken from skin swabs with confirmed genotypes; horizontal bars indicate 95% bootstrap confidence intervals for the median (B = 2000 resamples). The dashed vertical line marks zero bias. SNPs were retained for downstream analyses when their entire 95% CI lay within ±0.05.
