# Supplementary material for: Hybrid Horizons: Screening Hybridisation Through Nuclear Environmental DNA
Source: Mol Ecol Resour. 2026 May 4;26:e70134. doi: 10.1111/1755-0998.70134 (PMC13137079; doi:10.1111/1755-0998.70134)
Supplement: Supplementary file 3 — Figure S3: Paired Bland–Altman comparing estimates from each sampling regime. Each point is a population sampled under both regimes, whose HI estimates are averaged across SNPs. Positive values indicate that low concentration (conc.) eDNA sampling yields higher HI than high conc. eDNA sampling for the same population; negative values indicate the opposite. Green solid line is the mean bias and red dashed lines the 95% limits of agreement (LoA = mean ±1.96 SD). Mean bias was 0.021 with LoA [−0.191, 0.148], indicating minimal average discrepancy between sampling regimes. [file MEN-26-e70134-s002.pdf]

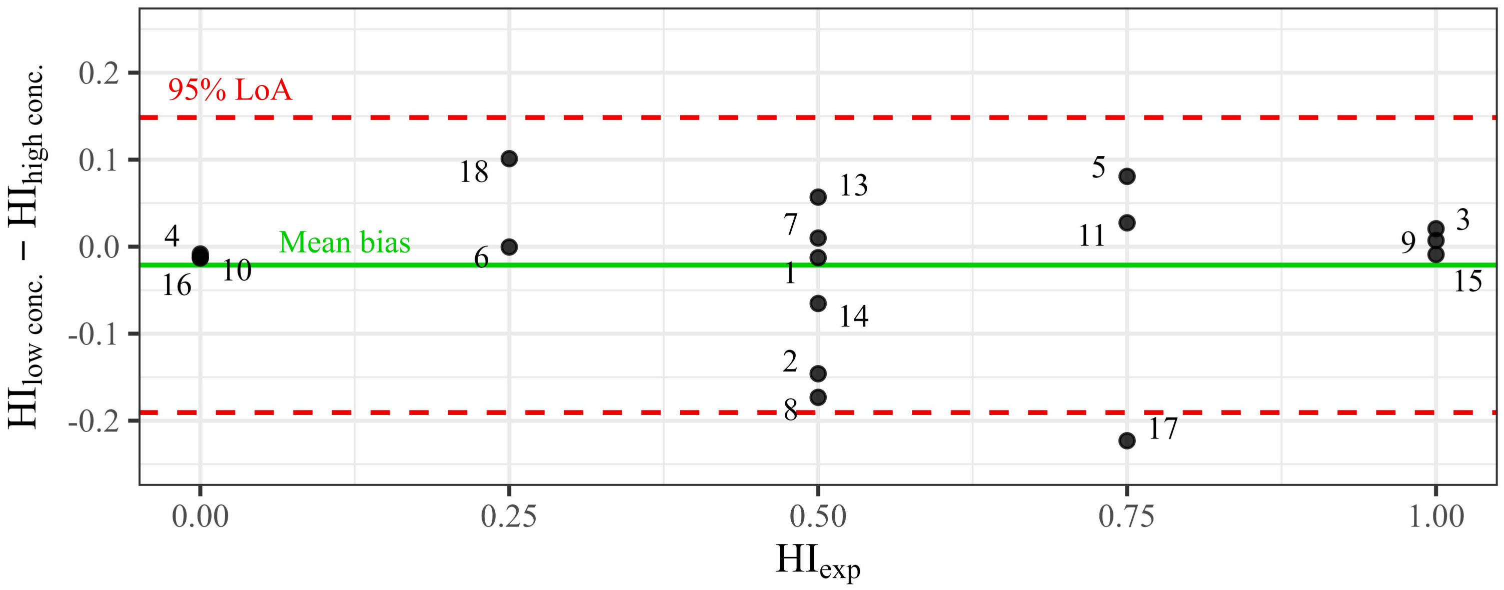

Figure S3. Paired Bland–Altman comparing estimates from each sampling regime. Each point is a population sampled under both regimes, whose HI estimates are averaged across SNPs. Positive values indicate that low concentration (conc.) eDNA sampling yields higher HI than high conc. eDNA sampling for the same population; negative values indicate the opposite. Green solid line is the mean bias and red dashed lines the 95% limits of agreement (LoA = mean ± 1.96 SD). Mean bias was 0.021 with LoA [−0.191, 0.148], indicating minimal average discrepancy between sampling regimes.
